# Impact of Next-Generation Computed Tomography Scanners on Image Quality and Radiation Dose in Cardiac CT Outpatient Imaging

**DOI:** 10.1055/a-2536-3534

**Published:** 2025-03-05

**Authors:** Henrik Jakob Michaely, Melanie Lueck, Miriam van Rickeln

**Affiliations:** 1 Radiology, MVZ Radiologie Karlsruhe, Karlsruhe, Germany

**Keywords:** heart, CT-angiography, cardiac ct, dual-source ct, image quality, radiation dose

## Abstract

**Purpose:**

Retrospective analysis of 245 consecutive cardiac CTA cases before and after transition from a 128-slice single-source CT scanner (SSCT, Definition AS+, temporal resolution 150ms, detector width 38.4mm) to a 128-slice dual-source CT scanner (DSCT, Pro.Pulse, 86ms, 38.4mm).

**Materials and Methods:**

A total of 113 patients (33f/80m, mean age 66.0 years) were examined using the SSCT scanner, while 132 patients (43f/89m, 64.7 years) were examined using the DSCT scanner. Protocol selection (sequential/spiral) was performed manually on the SSCT scanner. On the DSCT scanner, protocol selection was automated using built-in AI. Heart rate, kV for CTA, and DLP (mGy·cm) for CTA were recorded. Image quality was independently evaluated for the RCA, LM, LAD, and LCX by two experienced readers on a three-point ordinal scale: 3 – excellent, 2 – minor artifacts but diagnostic, 1 – artifacts rendering vessel evaluation incomplete.

**Results:**

The mean heart rate of the patients was not significantly different between the SSCT scanner (61.1 bpm) and DSCT scanner (61.6 bpm). The mean kV for CTA was 101.4 on the SSCT scanner and 74.2 on the DSCT scanner (p<0.0001). The mean radiation dose for cardiac CTA, measured as DLP, was 134.2 on the SSCT scanner, 53% higher than the 87.7 on the DSCT scanner (p=0.001). For the SSCT scanner, 32% and 37% of RCAs were non-diagnostic according to readers 1 and 2, compared to 3% and 2% for the DSCT scanner. For the LM, LAD, and LCX, the rate of non-diagnostic cases was 2–7% with the SSCT scanner and 0–1% with the DSCT scanner.

**Conclusion:**

Under otherwise unchanged external conditions, the switch from SSCT to DSCT scanner for cardiac CT resulted in a significant reduction in radiation dose by approximately 34%, along with a significant decrease in non-diagnostic examinations.

**Key Points:**

- Switching from an SSCT to a DSCT scanner in an outpatient setting results in a significant dose reduction of approximately 34%.
- The number of artifact-related non-diagnostic examinations is significantly reduced when using a DSCT scanner with higher temporal resolution.
- Outpatient cardiac CTA can be performed with an average radiation dose well below 2 mSv using both SSCT and DSCT systems.

**Citation Format:**

- Michaely HJ, Lueck M, van Rickeln M. Impact of Next-Generation Computed Tomography Scanners on Image Quality and Radiation Dose in Cardiac CT Outpatient Imaging. Rofo 2025; 197: 1433–1439

## Zusammenfassung

**Ziel:**

Untersuchung des Einflusses eines CTs neuerer Bauart mit Dual-Source-Technologie auf Bildqualität und Strahlendosis in der ambulanten Radiologie.

**Material und Methoden:**

Retrospektive Analyse von 245 konsekutiven Herz-CTA-Fällen vor und nach der Umstellung von einem 128-Zeilen Single-Source CT (SSCT, Definition AS+, zeitliche Auflösung 150ms, Detektorbreite 38,4mm) auf ein 128 Zeilen Dual-Source CT (DSCT, Pro.Pulse, 86ms, 38,4mm). 113 Patienten (33/80 f/m, durchschnittliches Alter 66,0 a) wurden auf dem SSCT untersucht, 132 Patienten (43/89 f/m, 64,7 a) wurden auf dem DSCT untersucht. Die Protokollwahl (Sequenz/Spirale) erfolgt auf dem SSCT manuell, bei dem DSCT über die eingebaute KI automatisch. Die Herzrate, kV für die CTA und DLP (mGycm) für die CTA, wurde aufgezeichnet. Die Bildqualität wurde von 2 erfahrenen Befundern für die Gefäße RCA, LM, LAD, LCX auf einer ordinalen 3-Punkt-Skala unabhängig bewertet: 3- exzellent, 2- geringe Artefakte aber diagnostisch, 1- Artefakte, Gefäß nicht komplett beurteilbar.

**Ergebnisse:**

Die mittlere Herzrate der Patienten war mit 61,1bpm (SSCT) beziehungsweise 61,6bpm (DSCT) nicht signifikant unterschiedlich. Die mittlere kV für die CTA war beim SSCT 101,4, bei DSCT 74,2 (p<0,0001). Die mittlere Dosis für die Herz-CTA auf dem SSCT war mit einem DLP von 134,2 53% höher als auf dem DSCT mit einem DLP von 87,7 (p= 0,001). Für das SSCT waren bei Reader 1 und 2 32% beziehungsweise 37% der RCA nicht diagnostisch, für das DSCT 3% beziehungsweise 2%. Bei der LM, LAD und LCX lag die Rate der nicht diagnostischen Fälle für das SSCT bei 2–7%, beim DSCT zwischen 0–1%.

**Schlussfolgerung:**

Bei sonst unveränderten äußeren Umständen führt allein die Umstellung von einem SSCT auf ein DSCT für die Herz-CT zu einer signifikanten Reduktion der Strahlendosis um circa 34% bei gleichzeitig signifikanter Verminderung nicht diagnostischer Untersuchungen.

**Kernaussagen:**

- Der Wechsel von einem SSCT auf ein DSCT im ambulanten Setting führt zu einer signifikanten Dosisreduktion von ca. 34%.
- Die Anzahl Artefakt-bedingter nicht-diagnostischer Untersuchungen ist bei Verwendung eines DSCT mit höherer zeitlicher Auflösung signifikant vermindert.
- Die ambulante Herz-CTA kann sowohl mit einem SSCT als auch einem DSCT mit einer durchschnittlichen Strahlendosis von deutlich unter zwei mSv durchgeführt werden.

## Introduction

Cardiac CT has become an established method for visualizing coronary vessels. The diagnostic accuracy has been reported in meta-analyses with a sensitivity and specificity of 95.2%/79.2% 1 2 . In the current “Nationale Versorgungsleitlinie Chronische KHK” (national care guidelines for chronic coronary artery disease), cardiac CT is recommended as the method of choice for patients with low to intermediate pretest probability 3 . It demonstrates identical accuracy compared to invasive coronary angiography but with significantly fewer side effects and leads to reduced mortality in the long term 4 5 . In Germany, the Bundesärztekammer (Federal Medical Association) regulates the minimum requirements for device technology for various indications, including cardiac CT, through guidelines for quality assurance in computed tomography 6 . Key parameters include coverage in the z-direction with a detector having at least 64 slices and a temporal resolution with a rotation time of ≤0.35s. This means that only mid-range or higher-priced CT scanners qualify for cardiac CT applications.

For many outpatient practices, the question arises as to whether the additional cost of a cardio-capable CT scanner is economically justifiable and what technical features the CT scanner should have. The answer to the first question depends on local circumstances, billing codes yet to be defined, quality assurance agreements, and respective regional regulations. This publication aims to help answer the second question.

For more than ten years, cardiac CT scans have been performed at our institution using a 128-slice single-source CT (SSCT) scanner with a rotation time of 0.3s. Annually, over 300 cases have been examined, with most patients referred by specialized cardiologists. In the context of a practice expansion, a new 128-slice dual-source CT (DSCT) scanner with a rotation time of 0.33s was installed in the summer of this year. From the moment of installation of the scanner, all cardiac CT patients have been exclusively examined on the new DSCT scanner. Referring physicians, patient quality, staff, and contrast media protocols have remained unchanged. This provided an opportunity to conduct a retrospective analysis of image and metadata to make a robust assessment of the impact of CT scanner technology on image quality and radiation dose in an outpatient setting. These data can serve as guidelines for other providers of CT services when selecting scanner technology.

## Materials and Methods

### CT Technology

Before the transition, a 128-slice single-source CT scanner (SOMATOM Definition AS+, Siemens Healthineers, Forchheim, Germany) with a native temporal resolution of 150ms (rotation time 0.3s) and a detector width of 38.4mm was used. After the transition, a 128-slice dual-source CT scanner (SOMATOM Pro.Pulse, Siemens Healthineers) with a native temporal resolution of 86ms (rotation time 0.33s) and a detector width of 38.4mm was employed. Further technical details of both CT scanners are listed in Table 1 .

**Table:** **Table 1** Comparison of the main image acquisition and reconstruction parameters for the SSCT and DSCT scanners.

	SSCT scanner	DSCT scanner
**Number of detector rows**	128	128
**Number of X-ray tubes**	1x Straton tube	2x Athlon tube
**Rotation time (s)**	0.3	0.33
**Temporal resolution (ms)**	150	86
**Detector width z-axis (mm)**	38.4	38.4
**Recon slice thickness (mm)**	0.75	0.8
**Recon method**	Iterative (SAFIRE)	Iterative (SAFIRE with ZeeFree)
**Recon kernel**	BV38	BV40
**Contrast media protocol**	50ml Imeron 400 @ 5ml/s 50ml Imeron 400/50ml Nacl @ 5ml/s 50ml NaCl @ 5ml/s	50ml Imeron 400 @ 5ml/s 50ml Imeron 400/50ml Nacl @ 5ml/s 50ml NaCl @ 5ml/s
**Protocol selection (sequence/spiral)**	Manual	Automatic

For both CT scanners, the manufacturer’s built-in dose modulation (Care-kV) was used for CT angiography. Contrast agent timing was performed via bolus triggering in the ascending aorta, with scan initiation occurring automatically upon reaching the threshold of 140HU. On the SSCT scanner, CT angiography was acquired using prospectively triggered sequences (up to a heart rate of 65 bpm). Above this threshold, retrospectively triggered spiral acquisition was used. For the prospective sequence, a diastolic acquisition window of 70–70% (low heart rate, stable) or 60–80% (low heart rate, instable) was used. The spiral acquisition for higher heart rates used the 30–40% cardiac cycle for the main dose. On the DSCT, program selection (sequence vs. spiral) was performed automatically via built-in AI, which chose the optimal program based on heart rate and rhythm. Typically, a sequence with an exposition window at 75–75% was used when a low and rhythmic heart rate was detected. With faster or arrhythmic heart rates, padding was added or a sequential systolic acquisition or if need be a pulsed spiral with the main dose applied at 30–40% of the cardiac cycle was chosen by the scanner.

Contrast agent was administered using a MedRad Stellant injector pump (Bayer, Leverkusen, Germany). A total of 50ml of Imeron 400 (Bracco, Milan, Italy) was followed by an Imeron/NaCl mixture (50ml/50ml) and 50ml NaCl at 5ml/s, delivered through a peripheral 18G venous cannula.

### Patients

The data for all cardiac CT patients examined within 80 days before and after the commissioning of the new CT scanner were retrospectively included. Image data were queried through the PACS (Phönix-PACS, Freiburg, Germany), which allows anonymized queries and data display. Exclusion criteria included patients undergoing coronary calcium measurement without CT angiography and patients with bypasses. In total, data from 245 patients were included.

### Image Analysis

From the CT angiography (CTA) protocols stored in the PACS, the dose-length product (DLP) for the CTA and the kV used for the CTA were extracted. The heart rate at the time of acquisition and the acquisition mode were also extracted. The image quality of the coronary vessels was independently evaluated by two experienced readers (Q2 and Q3 Cardiac CT of the Deutsche Röntgengesellschaft (German Radiological Society)) on a vessel basis for the RCA, LM, LAD, and LCX using an ordinal 3-point scale: 3 – excellent, 2 – artifacts but diagnostic, 1 – artifacts rendering the vessel not fully evaluable.

### Statistical Analysis

Using an alpha level of 0.05 and a power of 0.8, the given sample size would allow robust detection of small differences between both groups (Cohen’s d = 0.4). Data were anonymized and stored in Excel (Microsoft, Redmond, WA). Statistical analysis was performed using JASP (University of Amsterdam, Netherlands) and ChatGPT 4o (OpenAI, San Francisco, USA). Means, standard deviations, and two-sided Student’s t-tests were used for continuous data, while medians and Wilcoxon signed-ranks tests were employed for ordinal data. The agreement between readers was calculated using Cohen’s kappa coefficient.

## Results

A total of 113 patients (32 females/80 males, average age 66.1 years) were examined using the SSCT scanner, while 132 patients (43 females/89 males, average age 64.7 years) were examined using the DSCT scanner. The average age of patients and the female-to-male ratio were not significantly different between the two CT scanners. The mean heart rate was also not significantly different between the two scanners, averaging approximately 61–62 beats per minute. The variability in heart rate, which was partly very high on both systems due to extrasystoles during acquisition, was identical for both CT scanners. A detailed overview of the demographic data is provided in Table 2 .

**Table:** **Table 2** Patients’ demographic data and acquisition-related data comparison.

	SSCT scanner	DSCT scanner	
**Number of patients**	113	132	
**Gender (F/M)**	33/80	43/89	
**Age range (a)**	43–90	40–90	
**Mean age (a)**	66.0±10.5	64.7±10.5	p=0.3 (n.s.)
**Heart rate range (bpm)**	38–123	41–130	
**Mean heart rate (bpm)**	61.1±10.8	61.6±14.1	p=0.7 (n.s.)
**Sequence/spiral**	95/18 (84%/16%)	120/12 (91%/9%)	
**Mean CTA kV**	101.4±12.1	74.2±7.5	p<0.0001
**DLP range (mGy∙cm)**	27–923	17–558	
**Mean DLP CTA (mGy∙cm)**	134.2±138.4	87.7±65.9	p=0.001
**bpm – beats per minute**			

**Fig. 1:**
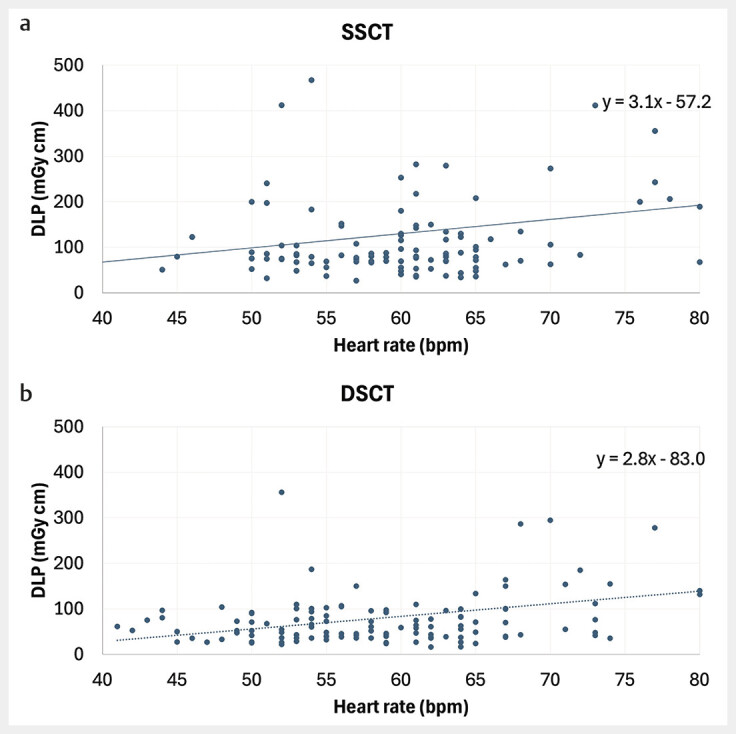
**a** DLP across different heart rates – SSCT scanner. **b** DLP across different heart rates – DSCT scanner. The distribution of DLP across different heart rates demonstrates significantly lower variability with the DSCT scanner. Additionally, DLP increases less steeply at higher heart rates on the DSCT scanner. The graph has been truncated for clarity, omitting high DLP values above 500 mGy · cm and heart rates above 80 bpm for both devices.

**Fig. 2:**
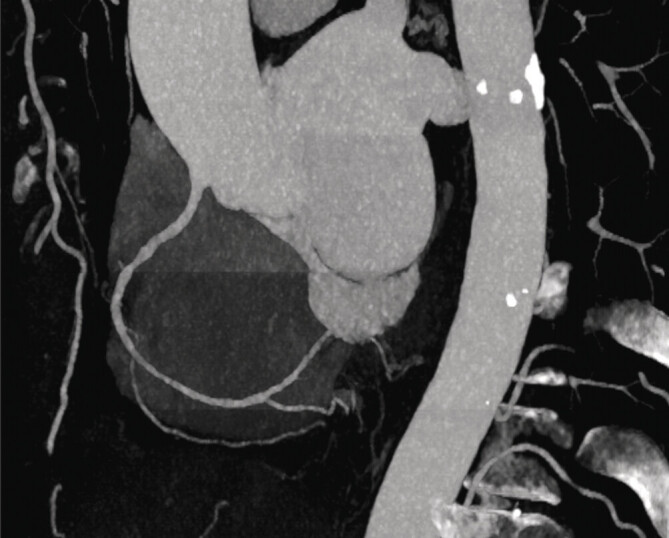
Exemplary depiction of the right coronary artery and its branches. This is the patient with the lowest dose of 0.24 mSv. The minimal differences in contrast, particularly in the right ventricle, are also clearly visible due to the sequential acquisition, without any visible steps in the RCA. The image quality was rated as very good (score 3) by both reviewers.

The mean DLP for the DSCT scanner was 87.7 mGy·cm, which was 52% lower than for the SSCT scanner at 133.6 mGy·cm (p=0.013). The DLP varied on both CT scanners depending on patient constitution and examination method, ranging from 17 mGy·cm to 923 mGy·cm. For the DSCT scanner, the DLP showed less variation across all heart rates ( Fig. 1 ). Using a conversion factor of 0.014, the mean effective radiation dose was calculated as 1.23 mSv for the DSCT scanner and 1.87 mSv for the SSCT scanner. An example of image quality is shown in Fig. 2 .

**Fig. 3:**
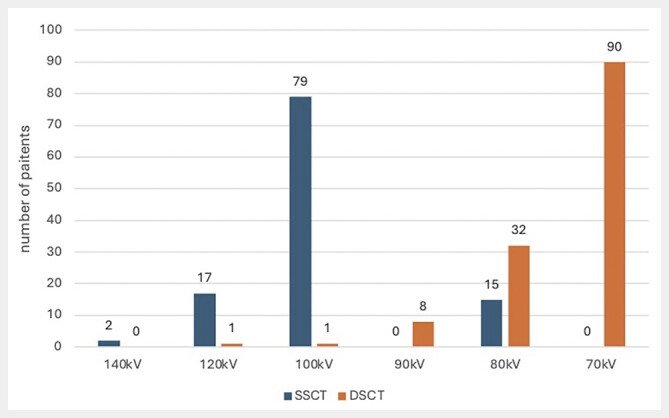
Representation of the kV distribution across different patients. In the case of the SSCT scanner, most patients were examined with 100 kV, with a few cases using 120 kV or 80 kV. In the case of the DSCT scanner, the stronger X-ray tube allowed for examinations predominantly at 70 kV and 80 kV.

The automatic kV selection allowed for a significantly lower average tube voltage on the DSCT scanner (74.2 kV) compared to the SSCT scanner (101.4 kV) (p<0.001). The distribution of kV across individual examinations is shown in Fig. 3 . The complete technical results are also provided in Table 2 .

**Fig. 4:**
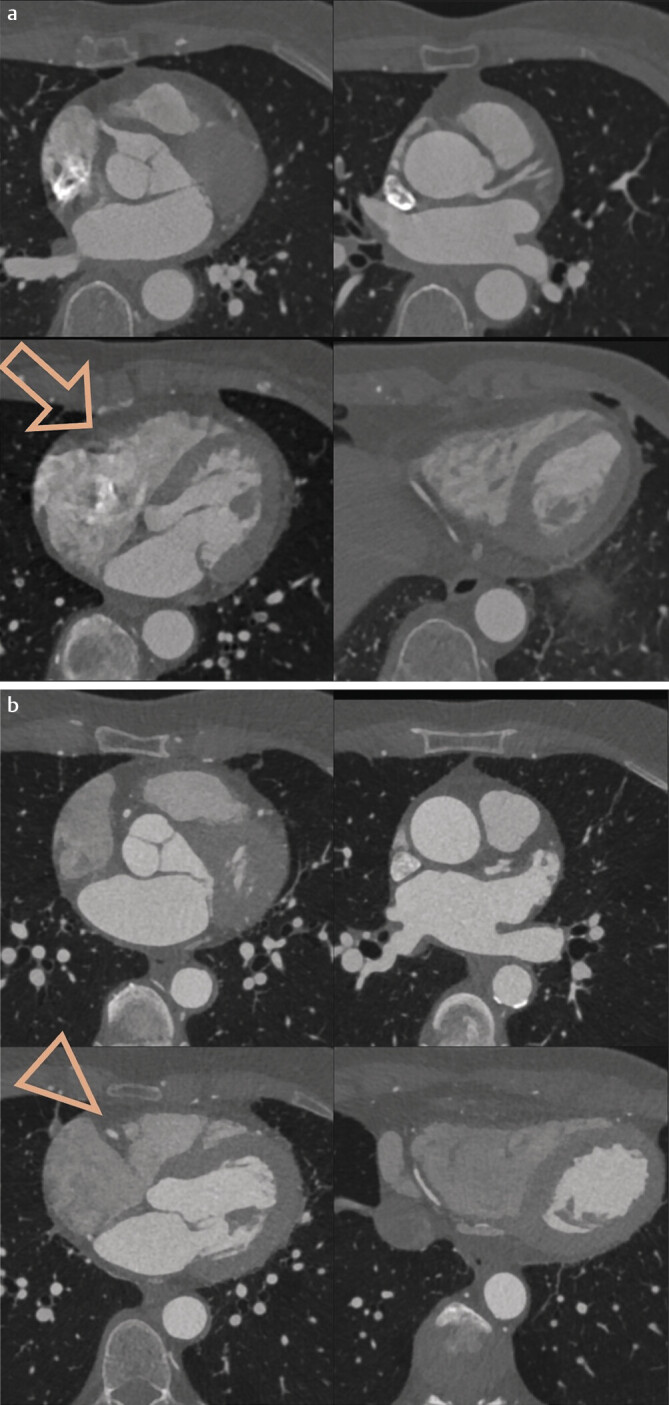
Comparable slices from an SSCT examination ( **a** ) and a DSCT examination ( **b** ). The LAD and distal RCA are clearly visible, but the mid-RCA in the sulcus is blurred due to pulsation in the SSCT examination (arrow) and cannot be assessed, whereas it is well-assessable in the DSCT examination (arrowhead). To better visualize the artifacts in the sulcus, a thin MIP was used for the SSCT examination in this representation.

In the subjective evaluation of vessel assessability by the two readers, diagnostic image quality of the right coronary artery (RCA) was achieved for the SSCT scanner in 37 of 113 cases and 42 of 113 cases (readers 1 and 2, respectively), as shown in Fig. 4 . Calculated as percentages, 33% and 37% of RCA examinations on the SSCT scanner were non-diagnostic (readers 1 and 2, respectively). For the DSCT scanner, the number of non-diagnostic RCAs was 8 and 5 of 132 cases (readers 1 and 2, respectively). In percentages, this corresponds to 6% and 4% non-diagnostic RCA examinations with the DSCT scanner.

For the circumflex artery (LCX), the percentage of non-diagnostic examinations with the SSCT scanner was 6% and 7% (readers 1 and 2, respectively), while for the DSCT, it was 2% and 0% (readers 1 and 2, respectively). The percentage of non-diagnostic left anterior descending artery (LAD) examinations was 4% with the SSCT scanner (readers 1 and 2, respectively) and 0% with the DSCT scanner (readers 1 and 2, respectively). These differences were statistically significant: RCA p<0.0001, LCX p<0.0001, LAD p=0.0004. No relevant differences were found for the left main artery (LM).

The agreement between readers was good, with a Cohen’s kappa of 0.71 (0.61–0.8 – good agreement). A summary of the results of the subjective evaluation is presented in Table 3 .

**Table:** **Table 3** Display of the results of the vessel assessment for both readers where 1 indicates a non-diagnostic vessel/vessel segment, 2 indicates impaired but diagnostic image quality, and 3 indicates excellent image quality.

		Reader 1				Reader 2			
	Score	RCA	LM	LAD	LCX	RCA	LM	LAD	LCX
**SSCT**	1	37	2	5	7	42	2	4	8
	2	31	8	39	24	31	8	33	22
	3	45	103	69	82	40	103	76	82
**DSCT**	1	8	0	0	3	5	0	0	0
	2	33	1	24	13	32	1	11	16
	3	91	131	108	116	95	131	121	115

## Discussion

This study provides two key insights: first, the improved technology of newer CT devices, such as the dual-source CT (DSCT) scanner used in this study, leads to significantly more frequent utilization of “low-kV” imaging, which is particularly dose-efficient. As this method utilizes radiation closer to the k-edge of iodine, it achieves a high arterial vessel contrast on average 7 . However, measuring contrast was outside the focus of this study.

Second, the data clearly show that increased temporal resolution, as achieved with a DSCT scanner, results in a much more robust execution of cardiac CT in routine clinical practice. Even with the new DSCT scanner, non-diagnostic cases were observed, but the number of such cases was significantly lower compared to the single-source CT (SSCT) scanner. In particular, the relatively frequent pulsation artifacts of the RCA in the atrioventricular sulcus with the SSCT scanner were, while not completely eliminated with the DSCT scanner, significantly less frequent and pronounced. This resulted in a statistically significant improvement in the overall evaluability of the examinations, making routine practice examinations considerably more robust.

Translated into a radiological report, this means that out of the 113 cardiac CT examinations conducted on an SSCT scanner, depending on the reader, 37–42% would have to be rated as CAD-RADS N because not all segments were reliably evaluable. With the DSCT scanner, this percentage decreases to 4–6%. In practice, radiologists often resort to providing a summary evaluation of visible segments. However, a secure and comprehensive assessment of coronary vessels is desirable.

With the imminent rollout of cardiac CT as a service covered by statutory health insurance in Germany, an increasing number of examinations can be expected. To ensure that the method, which has so far been predominantly used in academic and university settings, gains acceptance in the private cardiology sector, consistently high radiological process quality and thus consistently good evaluability of coronary vessels are a fundamental requirement.

The minimum technical standards for cardiac CT set forth by the Federal Joint Committee (Gemeinsamer Bundesausschuss, GBA) and outlined in the Federal Medical Association’s guidelines 6 would be fully met with the SSCT scanner used in this study. The SSCT scanner that was used even had a slightly better rotation time (0.3s) than the legally required minimum standard (≤0.35s). However, given the results presented in this study, the technical minimum requirements mandated by law should be critically re-evaluated.

Of course, the successful performance of cardiac CT also requires proper patient premedication, including beta blockers if necessary, unless contraindicated. This was and is routinely done at our institution. However, not all patients can achieve the desired heart rate of below 60 bpm, and some may exhibit higher heart rates or even extrasystoles during acquisition due to the stress of the cardiac CT examination.

Patients with extrasystoles during acquisition or arrhythmias were also those who, along with individuals with higher body weight and compact body structures, showed significant outliers in radiation dose on both systems. The difference between the lowest and highest doses on the DSCT scanner was nearly a factor of 33: DLP ranged from 17 mGy·cm to 558 mGy·cm (0.24 mSv to 7.8 mSv). Similar differences were also observed with the SSCT scanner.

On average, the effective radiation dose on both systems was significantly lower than the previously published data, at 1.23 mSv (DSCT) and 1.87 mSv (SSCT). A recent review article published in the German Medical Journal reported the dose for prospectively triggered coronary CTA as 5.7 mSv 8 . The doses presented in this study are thus approximately 70–80% lower than published values.

This is particularly important given the high visibility and health policy impact of the German Medical Journal in Germany. However, it should be noted that the dose values reported here do not include the topogram and the dose for bolus tracking. Including these would increase the dose for both devices by approximately 0.2–0.5 mSv. For conventional coronary angiography, the reference value for a purely diagnostic coronary angiography is listed at 2800 cGy·cm², or approximately 4.48 mSv 9 —thus about 2–3 times higher than the effective doses for cardiac CT presented here.

These positive data must be proactively communicated by radiologists to referring colleagues to increase the acceptance of cardiac CT and highlight its added value compared to diagnostic coronary angiography.

It is also notable that the use of automated software for program selection on the CT scanner, as with the DSCT scanner, reduced the use of spiral acquisition from 16% of cases to 9%. This automation can also lead to higher process quality and reduce the workload for radiology technologists. It should also be mentioned that spiral acquisition does not automatically imply a high radiation dose: spiral acquisitions with DLPs as low as 73 mGy·cm (1.0 mSv) were found in this study for heart rates of 82 bpm. A systematic analysis of these cases, however, falls outside the scope of this study.

In summary, this practical, retrospective analysis of 245 cardiac CT cases shows that using the minimum technical requirements for cardiac CT examination (64-slice SSCT scanner, 0.35s rotation time) leads to a significantly higher rate of non-diagnostic findings compared to newer systems with higher temporal resolution. The radiation dose required for cardiac CT examination is significantly lower with both systems than in previously published data, with the DSCT scanner demonstrating nearly 34% lower doses than the SSCT scanner.

## Clinical Relevance

- Switching from an SSCT scanner to a DSCT scanner in an outpatient setting results in a significant dose reduction of approximately 34%.
- The number of artifact-related non-diagnostic examinations is significantly higher with an SSCT scanner (0.3s rotation time) compared to a DSCT scanner (0.33s rotation time).
- Meeting only the minimum standards for cardiac CT (64-slice SSCT scanner, 0.35s rotation time) is likely to result in a similarly high rate of non-diagnostic examinations as found in this study for the 128-slice SSCT scanner with a 0.3s rotation time.
- Outpatient cardiac CTA can be performed with an average radiation dose well below 2 mSv using both SSCT and DSCT systems.
